# Long non-coding RNA AOC4P suppresses hepatocellular carcinoma metastasis by enhancing vimentin degradation and inhibiting epithelial-mesenchymal transition

**DOI:** 10.18632/oncotarget.4344

**Published:** 2015-06-23

**Authors:** Tong-Hong Wang, Yong-Shiang Lin, Ying Chen, Chau-Ting Yeh, Yen-lin Huang, Tsung-Han Hsieh, Tzong-Ming Shieh, Chuen Hsueh, Tse-Ching Chen

**Affiliations:** ^1^ Tissue Bank, Chang Gung Memorial Hospital, Tao-Yuan, Taiwan; ^2^ Department of Anatomic Pathology, Chang Gung Memorial Hospital, Chang Gung University School of Medicine, Tao-Yuan, Taiwan; ^3^ Department of Hepato-Gastroenterology, Liver Research Center, Chang Gung Memorial Hospital, Tao-Yuan, Taiwan; ^4^ Department of Dental Hygiene, College of Health Care, China Medical University, Taichung, Taiwan

**Keywords:** long non-coding RNA (lncRNA), hepatocellular carcinoma (HCC), epithelial-mesenchymal transition (EMT), metastasis

## Abstract

Increasing evidence indicates that long non-coding RNAs (lncRNAs) regulate diverse cellular processes, including cell growth, differentiation, apoptosis, and cancer progression. However, the function of lncRNAs in the progression of hepatocellular carcinoma (HCC) remains largely unknown. We performed a comprehensive microarray analysis of lncRNA expression in human HCC samples. After validation in 108 HCC specimens, we identified a differentially expressed novel tumor suppressive lncRNA termed amine oxidase, copper containing 4, pseudogene (AOC4P). The level of AOC4P expression was significantly downregulated in 68% of HCC samples and negatively correlated with advanced clinical stage, capsule invasion and vessel invasion. Low AOC4P expression correlated with poor prognostic outcomes, serving as an independent prognostic factor for HCC. *In vitro* functional assays indicated that AOC4P overexpression significantly reduced cell proliferation, migration and invasion by inhibiting the epithelial-mesenchymal transition (EMT). RNA immunoprecipitation assays demonstrated that AOC4P binds to vimentin and promotes its degradation. Animal model experiments confirmed the ability of AOC4P to suppress tumor growth and metastasis. Taken together, our findings suggest that AOC4P lncRNA acts as an HCC tumor suppressor by enhancing vimentin degradation and suppressing the EMT. By clarifying the mechanisms underlying HCC progression, these findings promote the development of novel therapeutic strategies for HCC.

## INTRODUCTION

HCC is the fifth most common cancer and the third most common cause of cancer-related death worldwide. HCC is also the second most life-threatening malignancy among both males and females in Taiwan [1]. Based on recent estimates, approximately 8,000 new cases of HCC are diagnosed in Taiwan every year. Unfortunately, chemotherapy and surgical resection are not effective for patients with advanced HCC due to tumor recurrence, metastasis, and poor response to chemotherapy and radiotherapy [2–4]. Thus, the identification of novel genetic targets is crucial for HCC treatment.

In the human genome, approximately 2% of transcripts can be translated into proteins, whereas 98% of transcripts are noncoding RNAs (ncRNAs). ncRNAs can be broadly categorized into two groups: short and long non-coding RNAs (lncRNAs) [5]. Short ncRNAs are less than 200 nucleotides in length and include small interfering RNA (siRNA, 21–25 bp), piwi-associated RNA (piRNA, 23–30 bp), and microRNA (miRNA, 21–25 bp) [5, 6]. lncRNAs are between 200 bp and 100 kb in length and include ribosomal RNAs, transcribed pseudogenes, mRNA-like transcripts, and intronic transcripts. Recent studies have indicated that ncRNAs regulate various physiologic functions, including development, proliferation, migration, and apoptosis [5, 7]. For example, miRNAs decrease gene expression by targeting the 3′-UTR of mRNAs, inhibiting mRNA translation. Approximately 30% of human genes are regulated by miRNA, and miRNA deregulation is associated with several types of cancer [7–11]. In addition to miRNAs, thousands of lncRNAs were recently discovered via chromatin signature analysis and large-scale sequencing. The abnormal expression of lncRNAs is frequently observed in several cancer types [7, 12]. However, the biological function and clinical significance of lncRNAs in cancer remain largely unexplored.

Recent studies have estimated the number of lncRNAs to be approximately 15, 000. Additionally, most lncRNAs display a tissue-specific expression pattern. An increasing number of reports have indicated that lncRNAs function in a variety of biological processes and that the deregulation of lncRNAs correlates with many human diseases, including cancer, Alzheimer's disease, and heart disease [13–17]. To date, however, few lncRNAs have been associated with human cancer progression [18, 19]. Several lncRNAs have been used as diagnostic and prognostic markers, including the following: MALAT-1 for liver, lung, breast, and prostate cancer [20–24]; HOTAIR for breast, brain, and colon cancer [25–28]; OOC1 for colon cancer; and PCA3, PCAT-1, and SChLAP1 for prostate cancer [29–31]. The development of lncRNA microarrays has provided a high-throughput screening tool for lncRNA research and has facilitated the more thorough investigation of the clinical importance of lncRNAs. Moreover, the increasing number of lncRNAs that have been identified as being involved in cancer indicates the potential of lncRNAs for clinical applications.

Although thousands of lncRNAs have recently been identified, investigations regarding their functional roles in HCC remain limited. In this study, we identified a novel tumor suppressor lncRNA, termed amine oxidase, copper containing 4, pseudogene (AOC4P), via microarray analysis and subsequent validation in HCC tissue specimens. The expression of AOC4P was significantly downregulated in HCC tissues compared with normal adjacent tissues, and low AOC4P expression correlated with poor patient prognosis. Furthermore, *in vitro* functional assays indicated that AOC4P expression significantly reduced cell proliferation, migration and invasiveness by inhibiting the epithelial-mesenchymal transition (EMT). Using an *in vivo* animal model, we demonstrated that AOC4P exerts a tumor-suppressive effect by reducing tumor growth and metastasis. The findings of this study contribute to our understanding of the mechanism underlying HCC progression and enable the development of new therapeutic strategies for HCC.

## RESULTS

### Identification of lncRNAs that are deregulated in HCC tissues

To identify lncRNAs that are involved in the progression of HCC, total RNA was isolated from HCC samples obtained from 3 patients and matched normal adjacent tissues and was subjected to microarray analysis (Affymetrix GeneChip^®^ Human Gene 2.0). A fold–change of >2 was set as the threshold for gene expression differences. Using this threshold, we detected 41 upregulated and 43 downregulated lncRNAs in the HCC samples compared with the corresponding non-cancerous samples. To validate these findings, we selected 10 lncRNAs displaying a fold-change in expression of >4 and analyzed their expression via real-time polymerase chain reaction (PCR) in 15 paired HCC and adjacent non-cancerous tissues. The lncRNA AOC4P was identified as the most frequently downregulated lncRNA in our HCC samples.

### AOC4P is downregulated in HCC samples

**Figure 1:**
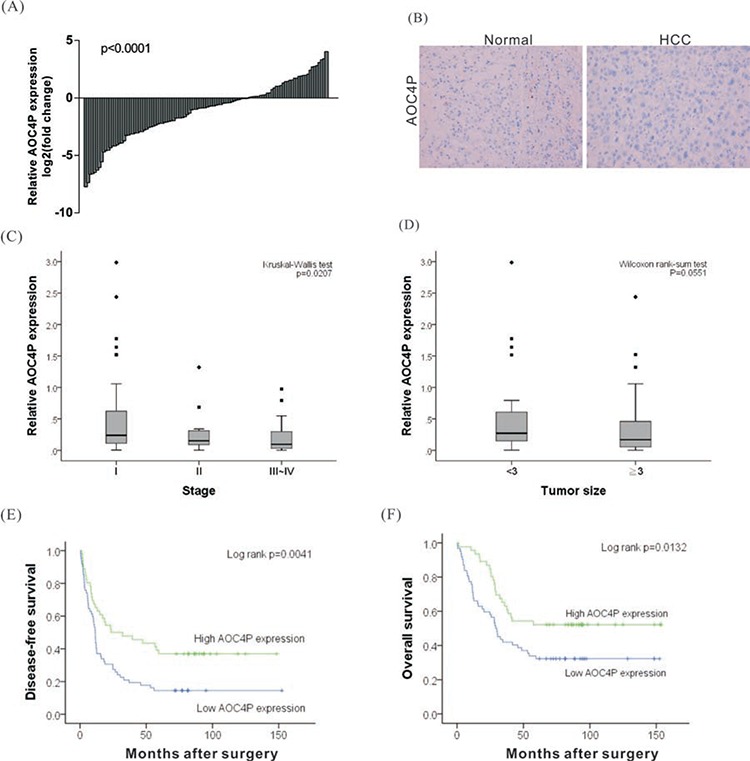
Relative expression of the lncRNA AOC4P in HCCs and its clinical significance **A.** The expression of AOC4P was examined in 108 paired human HCC and noncancerous normal tissues via qRT-PCR. GAPDH expression was assessed as an internal control. The results were presented as the fold-change in AOC4P expression tumor tissues relative to that in normal tissues. The expression of AOC4P was significantly lower in tumor tissues than in normal tissues (*p* < 0.001). **B.** *In situ* hybridization for AOC4P in HCC and in noncancerous normal tissues. AOC4P expression was notably reduced in HCC samples. **C and D.** AOC4P expression was significantly lower in patients with more advanced pathological stage tumors and with larger tumor size. **E and F.** Kaplan-Meier curve presenting the disease-free survival and the overall survival of HCC patients exhibiting high or low AOC4P expression. Lower AOC4P expression levels (relative expression level < 0.25) in the tumor tissues were associated with a poor prognostic outcome.

To analyze the biological significance of AOC4P downregulation to HCC, the levels of AOC4P were analyzed in 108 HCC tumor and paired non-cancerous tissues via quantitative real time RT-PCR. The relative expression of AOC4P in HCC tissues compared with normal tissues is shown in Figure 1A. Compared with normal liver tissue, the AOC4P expression level was significantly decreased in 68% of HCC tissue samples (73/108), by 10%–90%. To further evaluate the expression of AOC4P, an *in situ* hybridization assay was conducted. Consistently, significant downregulation of AOC4P expression was observed in HCC tissue (Figure 1B). Furthermore, a correlation analysis of AOC4P expression with clinicopathological parameters revealed that the AOC4P expression level was predominantly decreased in late-stage tumor tissues (Figure 1C, Kruskal-Wallis test *p* = 0.0207) and negatively correlated with tumor size (Figure 1D, Wilcoxon rank-sum test *p* = 0.0551). The downregulation of AOC4P in HCC suggested its potential function as a tumor suppressor.

### The downregulation of AOC4P is associated with a poor prognosis of HCC

To determine the clinical significance of AOC4P expression to HCC, we performed linear regression analysis using 108 HCC samples. The examined clinical characteristics of the patients included the following: gender, age, HBV or HCV carrier status, the presence of alcoholic liver disease, the degree of vascular invasion, capsule invasion and ascites formation, pathological stage, tumor size, and the levels of alpha-fetoprotein (AFP), albumin (Alb), bilirubin, creatinine (Cr), aspartate aminotransferase (AST), and alanine aminotransferase (ALT). These characteristics of the recruited HCC cohort are summarized in Table 1. Based on univariate analysis, the expression of AOC4P negatively correlated with smoking, capsule invasion, vessel invasion and pathological stage. No significant associations of AOC4P expression with other clinical or pathological parameters were found (Table 2). We further examined whether the AOC4P expression level correlated with survival outcomes of patients with HCC after surgery. Kaplan-Meier survival analysis followed by the log-rank test showed that lower AOC4P expression (relative expression level < 0.25) in tumor tissues correlated with reduced disease-free survival (*p* = 0.0041) and overall survival (*p* = 0.0132) among this HCC cohort (Figures 1E and 1F). In addition, vessel invasion, the serum albumin (Alb) level and capsule invasion were identified as significant prognostic factors (Table 3).

**Table 1:** Clinical parameters of the patients with HCC who were included in this study

Clinical parameters	AOC4P low (<0.25)	AOC4P high (≧0.25)
	*N* (%)	*N* (%)
Total number of patients	62	46
Gender		
male	51 (82.3)	42 (91.3)
Female	11 (17.7)	4 (8.7)
Age		
<55	27 (43.6)	19 (41.3)
≧55	35 (56.5)	27 (58.7)
Smoking		
Negative	30 (48.4)	28 (68.3)
Positive	32 (51.6)	13 (31.7)
Alcoholism		
Negative	33 (61.1)	30 (68.2)
Positive	21 (38.9)	14 (31.8)
HBV		
Negative	3 (4.8)	2 (4.4)
Positive	59 (95.2)	43 (95.6)
HCV		
Negative	1 (1.6)	2 (4.4)
Positive	61 (98.4)	44 (95.7)
Bilirubin		
<0.9	27 (43.6)	20 (43.5)
≧0.9	35 (56.5)	26 (56.5)
AST		
<52	34 (56.7)	37 (82.2)
≧52	26 (43.3)	8 (17.8)
ALT		
<111	48 (82.8)	38 (88.4)
≧111	10 (17.2)	5 (11.6)
Alb		
<4	25 (42.4)	18 (43.9)
≧4	34 (57.6)	23 (56.1)
Cr		
<1	22 (36.7)	13 (29.6)
≧1	38 (63.3)	31 (70.5)
AFP		
<10	13 (22.4)	15 (32.6)
≧10	45 (77.6)	31 (67.4)
Tumor size		
<3	15 (24.2)	19 (41.3)
≧3	47 (75.8)	27 (58.7)
Capsule invasion		
Absence	7 (11.3)	3 (6.5)
Presence	55 (88.7)	43 (93.5)
Vessel invasion		
Absence	25 (40.3)	26 (56.5)
Presence	37 (59.7)	20 (43.5)
Stage		
<2	36 (58.1)	34 (73.9)
≧2	26 (41.9)	12 (26.1)

**Table 2:** Regression analysis of AOC4P in relation to various clinical parameters

Clinical parameters	AOC4P expression			
	β	95%CI		*p*-value
Gender (Female vs male)	−0.22	−0.49	0.05	0.117
Age (<55 vs ≧55)	0.00	−0.20	0.19	0.961
Smoking (Positive vs Negative)	−0.20	−0.39	−0.02	0.033*
Alcoholism (Positive vs Negative)	−0.10	−0.31	0.11	0.334
HBV (Negative vs Positive)	−0.11	−0.55	0.33	0.633
HCV (Negative vs Positive)	−0.05	−0.63	0.53	0.864
Bilirubin (<0.9 vs ≧0.9)	0.04	−0.15	0.24	0.646
AST (≧52 vs <52)	−0.09	−0.29	0.12	0.401
ALT (≧111 vs <111)	0.09	−0.19	0.36	0.531
Alb (≧4 vs <4)	−0.08	−0.28	0.12	0.438
Cr (<1 vs ≧1)	0.18	−0.02	0.39	0.077
AFP (<10 vs ≧10)	−0.02	−0.24	0.20	0.842
Tumor size (<3 vs ≧3)	0.17	−0.03	0.37	0.098
Capsule invasion (Presence vs Absence)	−0.42	−0.73	−0.10	0.010*
Vessel invasion (Presence vs Absence)	−0.24	−0.42	−0.05	0.012*
Stage (≧2 vs < 2)	−0.23	−0.42	−0.03	0.023*

**Table 3:** Associations between AOC4P expression, clinical parameters and disease-free survival/overall survival

Clinical parameters		Disease-free survival (months)				Overall survival (months)			
	*N*	Mean	95%CI		*p*-value^#^	Mean	95%CI		*p*-value^#^
AOC4P expression									
<0.25	62	23.79	16.03	31.56	0.004*	44.25	33.92	54.58	0.013*
≧0.25	46	46.63	34.07	59.18		64.84	52.81	76.86	
Gender									
male	93	31.68	24.01	39.36	0.103	50.72	42.27	59.17	0.255
Female	15	44.90	23.34	66.47		67.28	42.73	91.83	
Age									
<55	46	34.79	22.97	46.61	0.975	58.14	45.19	71.08	0.413
≧55	62	32.57	23.39	41.76		49.22	39.02	59.41	
Smoking									
Negative	58	35.27	26.11	44.43	0.102	53.35	43.53	63.17	0.451
Positive	45	28.75	16.81	40.69		51.13	36.75	65.52	
Alcoholism									
Negative	63	37.61	28.19	47.04	0.130	59.51	49.03	70.00	0.118
Positive	35	30.73	16.70	44.76		45.91	30.83	60.99	
HBV									
Negative	5	22.49	−16.84	61.81	0.625	40.09	−16.71	96.89	0.594
Positive	102	34.31	26.84	41.77		53.89	45.69	62.09	
HCV									
Negative	3	29.38	−76.81	135.58	0.910	58.72	−74.69	192.13	0.548
Positive	105	33.64	26.35	40.92		52.86	44.80	60.91	
Bilirubin									
<0.9	47	34.79	24.16	45.43	0.584	53.02	41.12	64.92	0.770
≧0.9	61	32.54	22.58	42.49		53.01	42.03	64.00	
AST									
<52	71	36.70	27.56	45.84	0.283	55.53	45.33	65.73	0.542
≧52	34	28.67	15.95	41.40		50.73	36.92	64.53	
ALT									
<111	86	35.74	27.26	44.22	0.450	53.85	44.51	63.19	0.819
≧111	15	26.04	9.37	42.71		55.55	35.04	76.06	
Alb									
<4	43	26.77	15.73	37.81	0.079	41.11	29.76	52.46	0.032*
≧4	57	37.95	27.66	48.23		62.00	50.17	73.84	
Cr									
<1	35	29.15	16.59	41.70	0.473	52.00	37.14	66.87	0.541
≧1	69	33.95	24.84	43.06		52.14	42.06	62.21	
AFP									
<10	28	46.33	29.79	62.86	0.127	63.82	47.16	80.49	0.155
≧10	76	29.80	21.68	37.92		50.78	41.35	60.21	
Tumor size									
<3	34	42.41	28.14	56.68	0.118	63.24	48.17	78.31	0.091
≧3	74	29.43	21.20	37.66		48.32	38.96	57.68	
Capsule invasion									
Absence	10	15.27	4.52	26.02	0.074	31.47	13.64	49.30	0.041*
Presence	98	35.38	27.62	43.15		55.22	46.70	63.74	
Vessel invasion									
Absence	51	38.97	29.23	48.72	0.026*	53.18	43.08	63.27	0.451
Presence	57	28.64	18.10	39.17		52.88	40.52	65.24	
Stage									
< 2	70	35.89	26.41	45.38	0.546	57.26	46.88	67.63	0.389
≧2	38	29.14	18.22	40.06		45.21	32.93	57.49	

# Log rank test.

### The downregulated expression of AOC4P is an independent prognostic factor for patients with HCC

To determine the potential independent predictors of postoperative survival, a stepwise multivariate Cox proportional hazard model was generated. Higher AOC4P expression in HCC tissues and capsule invasion were associated with a 0.59-fold and 0.44-fold reduced risk of death, respectively. In contrast, vessel invasion was associated with a 1.82-fold increased risk of disease recurrence. Based on overall survival analysis, only high AOC4P expression and Alb levels in HCC correlated with a reduced risk of death (0.49-fold and 0.56-fold, respectively; Table 4).

**Table 4:** Stepwise multivariate Cox proportional hazard model for independent predictors for postoperative survival

Factors	Mutivariate			
	HR	95%CI		*p*-value
Disease-free survival				
High AOC4P (≧0.25 vs <0.25)	0.59	0.37	0.94	0.027*
Capsule invasion (Presence vs Absence)	0.44	0.21	0.94	0.033*
Vessel invasion (Presence vs Absence)	1.82	1.12	2.98	0.016*
Overall survival				
High AOC4P (≧0.25 vs <0.25)	0.49	0.28	0.85	0.011*
Alb (≧4 vs <4)	0.56	0.33	0.93	0.026*

### Forced expression of AOC4P reduced cell proliferation and migration *in vitro*

**Figure 2:**
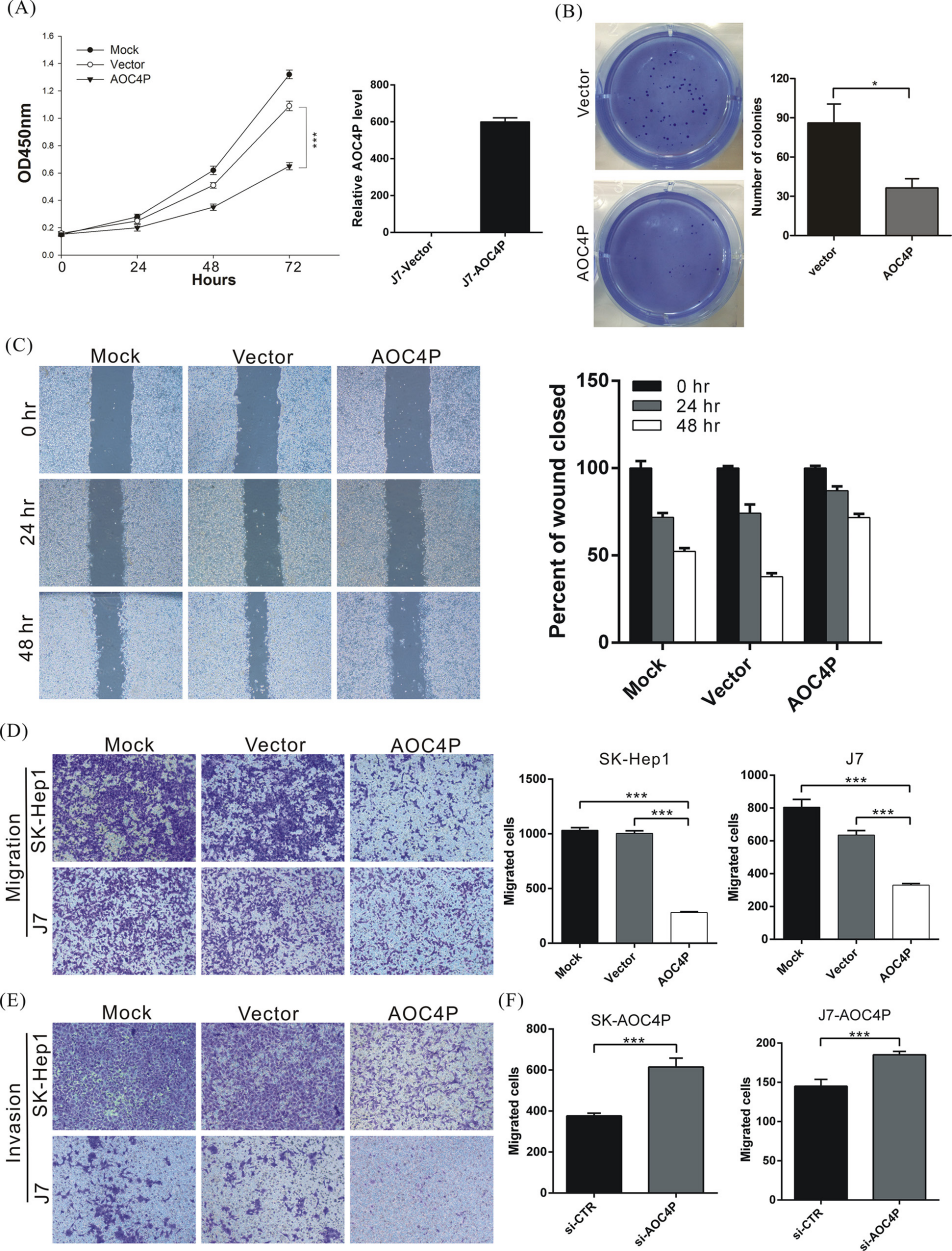
AOC4P suppresses the proliferation and migration of HCC cells *in vitro* **A.** J7 cells were transfected with the pCDNA3.1-AOC4P plasmid or empty vector, and then, cell proliferation was analyzed at the indicated time points via the MTT assay (left panel). The expression of AOC4P was also analyzed via qRT-PCR at 72 h after transfection (right panel). AOC4P overexpression significantly reduced cell growth 72 h compared with control vector transfection. Mock indicates no treatment. *p* < 0.001 (***). **B.** Colony formation ability was analyzed in cells at 12 days after transfection with the AOC4P expression plasmid or empty vector (left panel). The histogram shows that colony formation was significantly suppressed by AOC4P expression compared with empty vector transfection (right panel). **C.** The wound-healing assay results were compared between SK-Hep1 cells that were transfected with AOC4P plasmid or empty vector. More than 90% of the confluent cells were scraped using a 200 μl pipette tip and then photographed at 0, 24 and 48 h after scraping. The overexpression of AOC4P inhibited the wound-healing ability of SK-Hep1 cells. **D.** Cell migration was compared between J7 and SK-Hep1 cells that were transfected with either the AOC4P expression vector or empty vector. The overexpression of AOC4P significantly reduced cell migration ability (left panel). The quantification of the cell migration assays is presented (right panel). *p* < 0.001 (***). **E.** Invasion assays were performed using J7 and SK-Hep1 cells plated on Matrigel-coated polyethylene terephthalate membrane inserts. **F.** The migration activity of AOC4P -overexpressing cells was significantly rescued by treatment with AOC4P siRNA. The quantification of cell migration is presented. *p* < 0.001 (***).

To further examine whether AOC4P is involved in HCC progression, *in vitro* functional analyses were performed. AOC4P was overexpressed in cells, after which proliferation activity was analyzed via the MTT assay. As shown in Figure 2A, the proliferation activity of AOC4P-overexpressing cells was significantly suppressed at 72 h by approximately 44% compared with that in cells transfected with empty vector (*p* < 0.001***).

Cell motility, invasiveness and colony formation ability strongly correlate with cancer metastasis. Therefore, we examined whether AOC4P overexpression affects these characteristics of HCC cells. In Figure 2B–2E, we found that AOC4P overexpression significantly reduced HCC cell colony formation capacity and decreased their migration and invasion capabilities by approximately 40% and 60%, respectively. Conversely, the migration activity of AOC4P-overexpressing cells was significantly rescued by treating these cells with AOC4P siRNA (Figure 2F). These results suggest that AOC4P displays tumor suppressor activity that inhibits the progression of HCC.

### AOC4P binds to vimentin and enhances vimentin degradation

**Figure 3:**
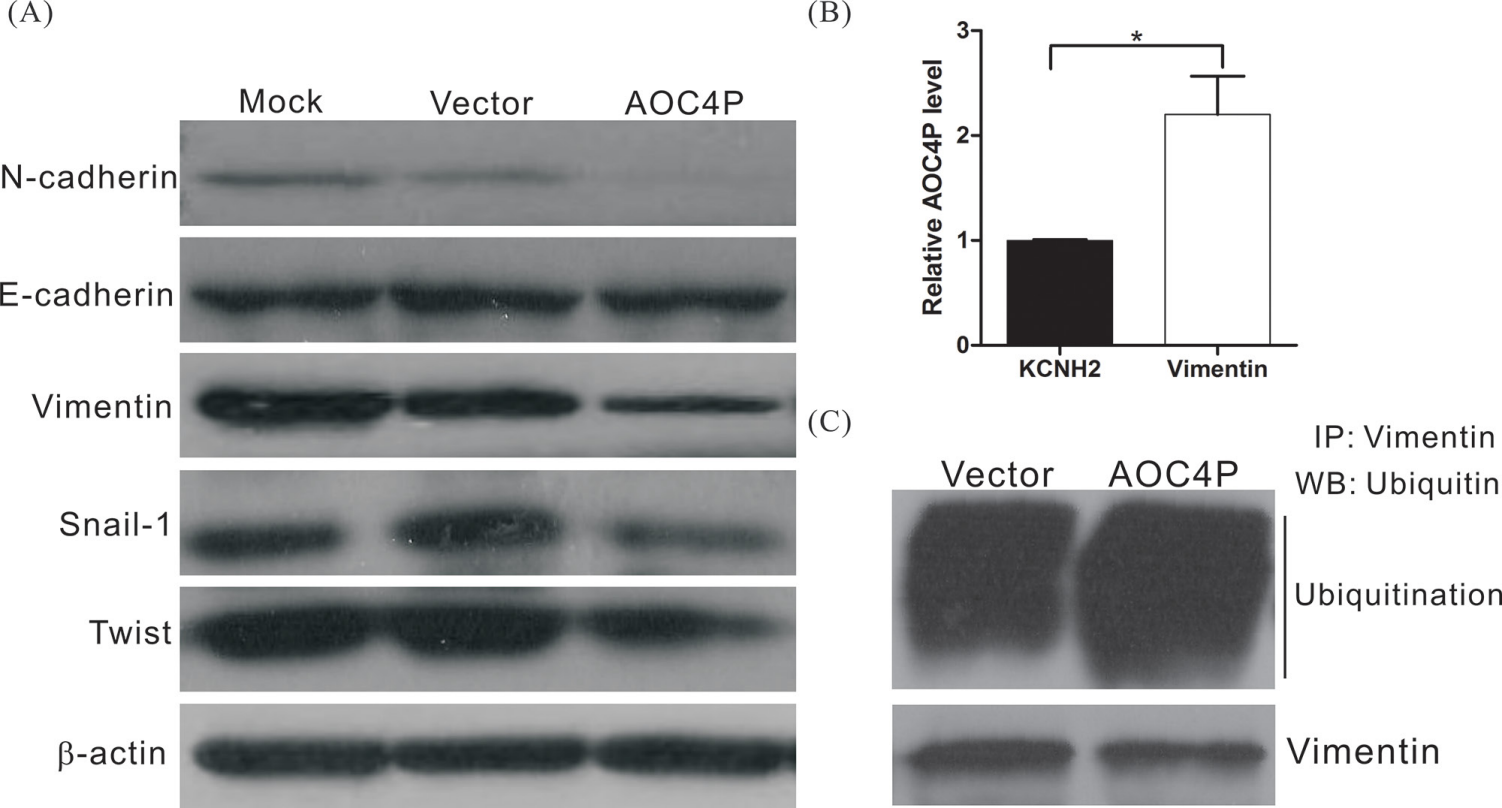
AOC4P binds to vimentin and enhances vimentin degradation **A.** Western blot analysis of EMT-associated proteins after transfection with the AOC4P expression plasmid or empty vector. β-actin expression was assessed as an internal control. **B.** RIP experiments were performed using SK-Hep1 cell lysates and an antibody against vimentin. An anti-KCNH2 antibody was included as a negative isotype control. The purified RNA was subjected to qRT-PCR for AOC4P detection. The results revealed that AOC4P binds to vimentin. **C.** Ubiquitination assay in SK-Hep1 cells that were transfected with the AOC4P expression plasmid or empty vector. AOC4P overexpression enhanced vimentin ubiquitination in SK-Hep1 cells.

During tumorigenesis, the EMT may increase the motility and invasiveness of cancer cells, and malignant transformation may involve signaling pathways that promote the EMT [32, 33]. To further investigate the possible mechanism by which AOC4P inhibits HCC progression, the expression levels of EMT-related proteins such as vimentin, N-cadherin, E-cadherin, snail and twist were examined. We found that the expression levels of vimentin, N-cadherin, snail and twist were significantly reduced in AOC4P-overexpressing cells, although no difference in E-cadherin expression was detected (Figure 3A). These results suggest that AOC4P suppresses HCC progression by inhibiting the EMT.

Recent studies have indicated that lncRNAs regulate a variety of biological processes by binding to proteins and altering their function [34, 35]. To further characterize the possible mechanism by which AOC4P inhibits the EMT, we performed an RNA pulldown assay followed by LC-MS analysis to identify AOC4P-associated proteins. Our results showed that several proteins, including vimentin, bind to AOC4P (Table 5). To further validate the binding between AOC4P and vimentin, an RNA immunoprecipitation (RIP) assay was performed using an antibody against vimentin. Consistent with our LC-MS results, we observed significantly higher enrichment of AOC4P using the anti-vimentin antibody when compared with a non-specific IgG control anti-KCNH2 antibody (Figure 3B). These results suggest that AOC4P binds to vimentin. We further examined whether AOC4P affects vimentin protein stability by performing a ubiquitination assay and found that the vimentin ubiquitination level was significantly higher in cells that overexpressed AOC4P relative to control cells (Figure 3C). These results suggest that AOC4P downregulates vimentin expression by enhancing vimentin degradation.

**Table 5:** Mass spectrometry analysis of the proteins pulled down by lncRNA-AOC4P

Hits	Description	protein score	protein mass (Da)	PSMs	peptides	protein coverage %
1	Heat shock protein HSP 90-beta	1790	83, 554	50	22	31.9
2	Heat shock cognate 71 kDa protein	1736	71, 082	46	18	32
3	60 kDa heat shock protein, mitochondrial	1633	61, 187	36	15	35.3
4	Pyruvate kinase PKM	1213	58, 470	25	11	28.4
5	Vimentin	449	53, 676	11	6	13.9
6	Proliferating cell nuclear antigen	439	29, 092	9	4	19.5
7	Elongation factor 1-alpha 1	383	50, 451	17	8	19
8	Malate dehydrogenase, mitochondrial	369	35, 937	11	5	20.7
9	Calmodulin	357	16, 827	8	2	22.1
10	Nucleolin	348	76, 625	10	7	9.3

### AOC4P suppresses tumor growth and metastasis *in vivo*

**Figure 4:**
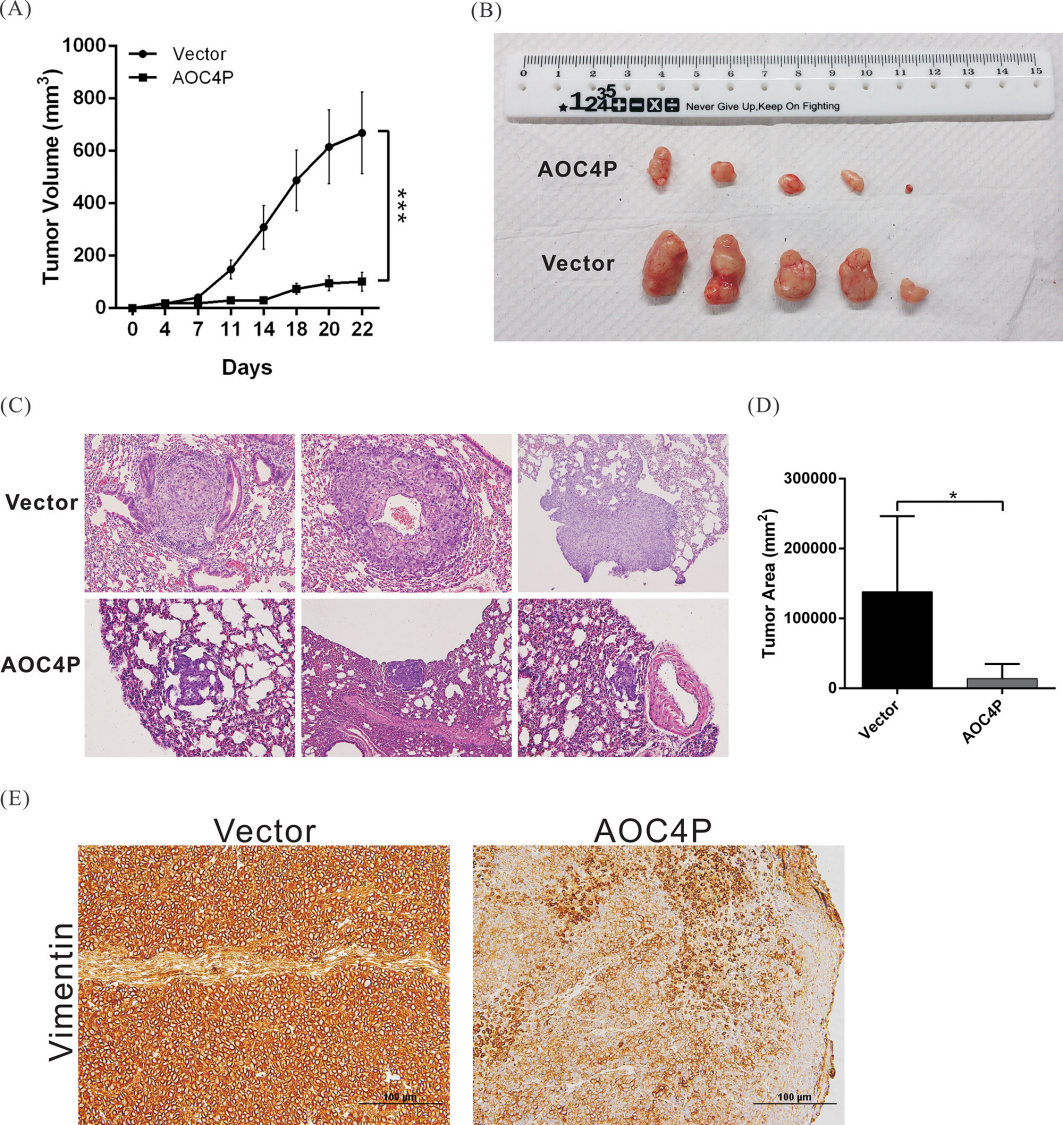
AOC4P inhibited HCC cell-based tumor growth and metastasis *in vivo* **A.** SK-Hep1 cells that were stably transfected with pCDNA3.1-AOC4P or with empty vector were implanted into nude mice (*n* = 5). The tumor volume was calculated every 3 days after implantation. The error bar indicates the S.D. ***p < 0.001. **B.** Representative images showing the tumor xenografts 4 weeks after implantation into nude mice. AOC4P expression significantly reduced tumor growth. **C.** Representative images of lung metastases based on H&E staining in nude mice 8 weeks after tail vein injection with SK-Hep1 cells that were stably transfected with pCDNA3.1-AOC4P or empty vector. AOC4P expression significantly reduced tumor metastasis. Magnification: 200×. **D.** Histological analysis of the lung tumor volume in the control group and the AOC4P overexpression group. The means ± S.D. are shown. (*n* = 5). *p < 0.05. **E.** Vimentin expression was lower in mice harboring grafts overexpressing AOC4P than in control mice harboring grafts expressing empty vector based on immunohistochemistry.

To further validate the tumor-suppressive function of AOC4P, we performed an *in vivo* xenograft assay and a tail vein migration assay using nude mice. Compared with the vector control, AOC4P expression significantly reduced tumor growth and lung metastasis (Figure 4A–4D). After 22 days of growth, the tumor volume was reduced in approximately 80% of mice harboring grafts overexpressing AOC4P compared with that in the mice of the vector control group. The suppression of vimentin expression in tumor tissue was also observed via immunohistochemical staining (Figure 4E).

## DISCUSSION

Hepatocellular carcinoma (HCC) is one of the most common malignant cancers worldwide and is the third leading cause of cancer-related death. Although clinical symptoms are not commonly observed during the early stages of HCC development, in most cases, the detection of symptoms during the advanced stage leads to a poor prognosis at the time of diagnosis. Thus, the exploration of new diagnostic and therapeutic molecular targets for HCC is particularly crucial. Recently, increasing reports have indicated the importance of lncRNAs in cancer progression; however, only a few reports have addressed the function of lncRNAs in HCC. In this study, we identified a novel lncRNA, AOC4P, and demonstrated its tumor-suppressive effect on HCC. We found that the expression of AOC4P was significantly downregulated in HCC specimens and that low AOC4P expression in HCC correlates with a poor prognostic outcome. Furthermore, both *in vitro* functional assays and an *in vivo* animal model demonstrated that AOC4P suppresses tumor growth by reducing cancer cell proliferation, migration and invasion ability at least in part by suppressing vimentin expression and the EMT.

Vimentin, the major component of the cytoskeleton, is overexpressed in mesenchymal cells. Vimentin is a well-known metastasis marker and therapeutic target, as inhibiting vimentin function reduces the ability of cells to migrate. Some anti-cancer drugs that are currently used in the clinic directly target vimentin. One such drug is silibinin, which inhibits HCC cell proliferation, migration, and invasion via the inhibition of vimentin expression and ERK signaling [36, 37]. Another example is withaferin A, a tumor inhibitor and antiangiogenic agent that targets the vimentin protein [38, 39]. In this study, we found that AOC4P downregulated vimentin expression, thereby reducing HCC tumor growth and metastasis. Our results suggest that the lncRNA AOC4P may represent a viable target for the development of anti-HCC drugs based on its ability to suppress vimentin expression.

Recent reports have indicated that lncRNAs are commonly expressed in a tissue-specific pattern [14, 40]. Accordingly, the transcription of lncRNAs must be tightly controlled. Similar to the expression of RNA polymerase II-transcribed protein-coding genes, the expression of lncRNAs can be regulated by transcriptional and epigenetic mechanisms. For instance, the expression of lncRNA-p21 is induced by the tumor suppressor protein p53 in response to DNA damage [41]. Moreover, MEG3 expression is indirectly promoted by mir-29a, which regulates the expression of methyltransferases, and the inhibition of DNA methyltransferase activity in HCC cells de-represses MEG3 expression [42]. The downregulation of AOC4P in HCC may be mediated by promoter methylation or by upstream transcription factor activity. Therefore, future investigation of the upstream regulatory machinery that acts on AOC4P in HCC could provide additional insight into HCC progression.

At present, tumor grade, tumor size, microscopic/macroscopic vascular invasion, and the alpha fetoprotein levels are considered to be important predictors of tumor recurrence. However, current clinical predictions based on clinicopathological characteristics remain unsatisfactory, and molecularly based tumor staging is essential for individualized diagnosis and therapy. Our findings suggest that HCC patients with tumors expressing high levels of AOC4P exhibit significantly increased overall postoperative survival and tumor-free survival rates. These findings support the concept that the AOC4P expression level could serve as a prognostic biomarker for HCC. Moreover, the AOC4P expression level in HCC could be used to predict the risk of disease recurrence and to guide the selection of therapeutic regimens.

In addition to AOC4P, we found 83 lncRNAs that are aberrantly expressed in HCC samples. The aberrant expression of lncRNAs indicates that lncRNAs may participate the development of HCC. The biological functions of these lncRNAs need to be investigated in greater detail to better understand their molecular mechanisms in HCC.

In conclusion, our data show that the lncRNA AOC4P exerts a tumor-suppressive effect on HCC tumor progression. To our knowledge, this is the first description of the function of the lncRNA AOC4P in HCC. Our results provide new insights into the function of lncRNAs in the development of HCC and suggest that AOC4P represents a potential therapeutic target and prognostic biomarker for HCC.

## MATERIALS AND METHODS

### Patients

The study cohort consisted of 108 patients with HCC who underwent surgical resection at Lin-Kou Chang Gung Memorial Hospital between 2000 and 2012. The clinical and pathological characteristics were obtained from patient charts. Tumors were staged according to the Seventh Edition of the Cancer Staging Manual by the American Joint Committee on Cancer, and the histological grade was scored according to the World Health Organization classification. This study was approved by the Ethics Committee of Chang Gung Memorial Hospital, and written informed consent was obtained from each patient.

### Detection of the lncRNA AOC4P in HCC samples via quantitative real-time RT-PCR

Paired HCC and non-cancerous tissues were obtained from the Tissue Bank of Lin-Kou Chang Gung Memorial Hospital. Total RNA from each tissue specimen was isolated using TRIzol reagent (Invitrogen, Carlsbad, CA, USA). To remove any contaminating genomic or plasmid DNA, the RNA samples were treated with RQ1 RNase-free DNase according to the manufacturer's instructions (Promega, Madison, WI, USA). Two micrograms of the treated RNA samples were subjected to quantitative real time RT-PCR to detect lncRNA-AOC4P expression using a TaqMan non-coding RNA assay (Applied Biosystems, Foster City, CA, USA); GAPDH was used as an internal control.

### Cell lines, antibodies, siRNAs and plasmid construction

The HCC cell lines J7 and SK-Hep1 were cultured in DMEM containing 10% fetal bovine serum at 37°C in an atmosphere containing 5% CO_2_. Polyclonal antibodies against E-cadherin, N-cadherin, vimentin, twist, snail and β-actin were purchased from Genetex (Irvine, CA, USA) or Santa Cruz Biotechnology (Santa Cruz, CA, USA). Secondary antibodies were purchased from Santa Cruz Biotechnology. All siRNAs were purchased from Applied Biosystems (Foster City, CA, USA). pCDNA3.1-AOC4P, a neomycin-selective CMV-based expression plasmid that contains the AOC4P lncRNA sequence, was constructed by GenScript (Piscataway, NJ, USA).

### Transient transfection of pCDNA3.1-AOC4P plasmids and Western blot analysis

Cell lines were seeded in 6-well plates at a density of 3 × 10^5^ cells/well and were cultured overnight. The cells were then transfected with 1 μg of either the pCDNA3.1-AOC4P or the pCDNA3.1 plasmid using Lipofectamine 2000 (Invitrogen, Carlsbad, CA, USA) according to the manufacturer's instructions. Forty-eight hours later, the transfected cells were washed twice with phosphate-buffered saline (PBS) and then lysed in 200 μl of RIPA lysis buffer (50 mM Tris–HCl, pH 7.4, 150 mM NaCl, 1 mM EDTA, 1% Triton X-100, 1% sodium deoxycholate, and 0.1% SDS) containing a protease inhibitor. Protein (100 μg) from the supernatants of the lysed cells was loaded on an SDS polyacrylamide gel for Western blot analysis to determine the expression levels of E-cadherin, N-cadherin, vimentin, twist, snail and β-actin. The immunoreactive bands were revealed using an electrochemiluminescence detection system (NEN Life Science Products, Boston, MA, USA) and were developed using x-ray film. The pixel intensity of each band was quantified using ImageQuant 5.2 software (GE Healthcare, Piscataway, NJ, USA).

### Cell proliferation assay

Cell growth was determined using the 3-(4, 5-dimethylthiazol-2-yl)-2, 5-diphenyltetrazolium bromide (MTT) assay as described in the product manual (Cayman Chemical, Michigan, USA). Briefly, cells were transfected with the pCDNA3.1-AOC4P or the pCDNA3.1 plasmid and then subjected to MTT analysis at 24, 48 and 72 hours after transfection to determine the cell proliferation rate.

For the colony formation assay, cells stably expressing AOC4P or carrying the empty vector were seeded on six-well plates at a density of 500 cells/well and maintained in DMEM containing 10% FBS for 12 days. The medium was replaced every 3 days. After 12 days, the colonies were fixed with methanol and stained with 0.1% crystal violet (Sigma–Aldrich, St. Louis, MO, USA). Visible colonies were photographed and counted manually.

### Cell migration and invasion assay

Cell migration was analyzed using a wound-healing assay and a Transwell migration assay. For the wound-healing assay, treated SK-Hep1 cells were plated in 6-well plates and cultured to 90% confluence. The cells were scraped using a 200 μl pipette tip (time 0), and the medium was replaced with low-serum culture medium. The migration distances of the cells were measured from the images (five fields) that were obtained at the indicated time points.

The migration and invasion abilities of J7 and SK-Hep1 cells were assessed using ThinCert Tissue Cell Culture Inserts (Greiner Bio-One, Kremsmunster, Austria) that contained a membrane with a mean pore size of 8 μm. For the migration assay, the cells were trypsinized and suspended in serum-free DMEM at a final concentration of 5 × 10^5^ cells/ml. The lower chambers were filled with 500 μl of complete medium (DMEM supplemented with 10% FBS), and 100 μl of cells were loaded in each upper well. The chambers were maintained in a *humidified incubator supplemented with 5% CO_2_* at 37°C for 24 hours. The cells were then fixed in 500 μl of methanol for 15 minutes, and the cells on the inner surface of the upper chambers were wiped with cotton swabs to remove the cells that had not migrated. The membrane was washed with 500 μl of PBS and stained with 500 μl of crystal violet for 20 minutes at room temperature. After the membranes were washed with 500 μl of PBS, the stained cells were imaged using ImagePro 6.2 software. Counts were obtained from five random fields at 100 × magnification. For the invasion assay, the membrane was coated with 30 mg/cm^2^ Matrigel (ECM gel, Sigma–Aldrich, St. Louis, MO, USA) to form a matrix barrier. The rest of the procedure was conducted in a manner identical to that of the migration assay, except that the cells were incubated on the Matrigel layer for 48 h.

### *In vitro* transcription and RNA pulldown assay

*In vitro* transcription was performed as described previously [43]. Briefly, the pCDNA3.1-AOC4P plasmid containing a T7 promoter was linearized via BamHI digestion, purified, and used as a template for *in vitro* transcription. The template was incubated in 1 mM each of ATP, CTP, GTP, UTP and T7 RNA polymerase in 1 × transcription buffer. The *in vitro*-transcribed RNA was purified using an RNeasy purification kit (Qiagen, Germany). The transcripts were labeled with biotin using a Thermo Scientific Pierce RNA 3′ Desthiobiotinylation Kit (Thermo Fisher Scientific, MA, USA). The RNA-protein binding reaction was performed using freshly harvested SK-Hep1 cells and the Pierce™ Magnetic RNA-Protein Pull-Down Kit (Thermo Fisher Scientific, MA, USA). The reaction products were then subjected to mass spectrometry for protein identification.

### Protein identification via mass spectrometry

After protein separation, the SDS-PAGE gel was stained with EZBlue (Sigma–Aldrich, St. Louis, MO, USA). The detected bands, along with a control region in which no proteins were detected, were excised. In-gel tryptic digestion was performed according to the manufacturer's protocol. The tryptic digest was then analyzed via mass spectrometry. Matrix-assisted laser desorption ionization–time of flight mass spectrometry was initially performed to identify peptide mass fingerprints. Specific peaks were selected for inclusion in tandem mass spectrometry analysis to identify the peptide sequences. Proteins were identified using MASCOT software.

### RNA immunoprecipitation assay

Protein-RNA complexes were immunoprecipitated using 3 μg of an anti-vimentin antibody; then, the RNA was purified using an RNeasy extraction kit (Qiagen, Hilden, Germany). The anti-KCNH2 antibody was used as a negative control. The extracted RNA was subjected to quantitative real-time RT-PCR to detect AOC4P lncRNA expression using a TaqMan non-coding RNA assay (Applied Biosystems, Foster City, CA, USA).

### Mice

Male 6- to 8-week-old BALB/C nude mice (purchased from the National Laboratory Animal Center, Taipei, Taiwan) were housed under pathogen-free conditions on a 12 h light/12 h dark schedule and were provided with free access to autoclaved standard chow and water. The mice were bred at the Animal Center of Chang Gung Memorial Hospital (Taoyuan, Taiwan) according to the Guidelines for the Care and Use of Laboratory Animals (NIH). All experiments related to the animal studies were approved by the Institutional Animal Care and Use Committee of Chang Gung Memorial Hospital.

### Tumorigenicity and *in vivo* metastasis assays

A total of 1 × 10^6^ J7 cells stably expressing the pCDNA3.1-AOC4P or the pCDNA3.1 plasmid were resuspended in 100 μl of saline containing 50% Matrigel (BD Biosciences) and were subcutaneously implanted into the left and right flank regions of the mice. The mice were anesthetized via intraperitoneal injection of a cocktail containing xylazine (80–120 mg/kg) and ketamine (10 mg/kg) to sedate the mice before tumor implantation. To measure tumor growth, the tumor volume was recorded weekly using digital calipers. For the *in vivo* metastasis assay, 1 × 10^6^ transfected SK-Hep1 cells were injected into the tail vein of nude mice. Eight weeks after SK-Hep1 cell implantation, the mice were sacrificed for analysis. Lung metastasis was examined via hematoxylin and eosin (H&E) staining, and the overall tumor burden was measured in five random fields at 100 × magnification.

### Immunohistochemistry

Tissues were fixed in formalin and embedded in paraffin, and 2-μm-thick consecutive sections were sliced and mounted on glass slides. The slides were first incubated at 65°C for 30 min and then subjected to deparaffinization in xylene followed by rehydration in a graded ethanol series. Then, the sections were boiled in Trilogy reagent (Cell Marque, Rocklin, CA, USA) for 10 minutes for antigen retrieval. After washing with 1 × PBS, the slides were immersed in 3% hydrogen peroxide for 10 min to suppress endogenous peroxidase activity. After three rinses with 1 × PBS, the sections were exposed to a mouse anti-vimentin antibody (Genetex, Irvine, CA, USA) for 1 hour at room temperature. After three rinses with 1 × PBS, the slides were incubated in a biotinylated secondary antibody (Dako, Glostrup, Denmark) for 25 min. The slides were then rinsed three times with 1 × PBS, followed by the addition of horseradish peroxidase (HRP)-conjugated streptavidin for 25 min at room temperature. Peroxidase activity was detected by incubating the slides in the chromogenic substrate 3, 3′-diaminobenzidine (DAB) (Dako) at room temperature. The slides were then counterstained with hematoxylin.

### LncRNA *in situ* hybridization

The expression and localization of the lncRNA AOC4P in tissues were analyzed using the RNAscope 2.0 FFPE Assay-Brown kit with custom-designed probes, according to the manufacturer's instructions *(Advanced Cell Diagnostics*, Inc., Hayward, CA). Briefly, paraffinized sections were incubated at 60°C for 1 h and then deparaffinized with xylene and 100% ethanol. After pretreatment, the sections were hybridized to target probes for 18 h at 40°C. The slides were then sequentially treated with Amp1 (preamplifier), Amp2 (signal enhancer), Amp3 (amplifier), Amp4 (probe labeler), Amp5 (signal amplifier) and Amp6 (HRP-linked labeling molecule). Following these amplification steps, the DAB substrate was added for the colorimetric detection of target RNA. Finally, the slides were counterstained with hematoxylin (Sigma–Aldrich, St. Louis, MO, USA) and mounted using whole-mount medium.

### Data analysis

The original real-time PCR data and the Western blot and migration assay data were recorded as continuous variables and were analyzed using Student's *t*-test. All statistical analyses were performed using SPSS 16.0 and Excel 2007 software. All statistical tests were two-sided, and the thresholds for significance were set at *P* < 0.05 (*), <0.01 (**), or <0.001 (***).
